# *Agaricus bisporus*-Derived Glucosamine Hydrochloride Regulates VEGF through BMP Signaling to Promote Zebrafish Vascular Development and Impairment Repair

**DOI:** 10.3390/life13122330

**Published:** 2023-12-12

**Authors:** Jiarui Sun, Qici Wu, Yuxin Wei, Wei Zhao, Haokun Lv, Wei Peng, Jiayi Zheng, Yixuan Chen, Zhengsen Wang, Yutian Pan, Yu Xue

**Affiliations:** 1 The Engineering Technological Center of Mushroom Industry, Minnan Normal University, Zhangzhou 363000, China; jiaruisun59@gmail.com (J.S.); wqc1806@mnnu.edu.cn (Q.W.); wyuxin54@gmail.com (Y.W.); weiz55749@gmail.com (W.Z.); haokunlv@gmail.com (H.L.); pwei7625@gmail.com (W.P.); zjoooooolly@gmail.com (J.Z.); cyx1883@mnnu.edu.cn (Y.C.); wzs2601@mnnu.edu.cn (Z.W.); 2 Fujian Fungal Active Substance Engineering Technology Center, Zhangzhou 363000, China

**Keywords:** glucosamine hydrochloride, vascular development, impairment repair, BMP, VEGF, zebrafish

## Abstract

Glucosamine hydrochloride (GAH) is a natural component of glycoproteins present in almost all human tissues and participates in the construction of human tissues and cell membranes. GAH has a wide range of biological activities, particularly in anti-inflammatory and osteogenic damage repair. At present, little is known about how GAH functions in angiogenesis. To determine the role of GAH on vascular development and impairment repair, we used the inhibitors VRI, DMH1, and dorsomorphin (DM) to construct vascular-impaired models in *Tg(kdrl: mCherry)* transgenic zebrafish. We then treated with GAH and measured its repair effects on vascular impairment through fluorescence intensity, mRNA, and protein expression levels of vascular-specific markers. Our results indicate that GAH promotes vascular development and repairs impairment by regulating the vascular endothelial growth factor (VEGF) signaling pathway through modulation of bone morphogenetic protein (BMP) signaling. This study provides an experimental basis for the development of GAH as a drug to repair vascular diseases.

## 1. Introduction

Glucosamine hydrochloride (GAH) is a basic derivative of the polysaccharides chitin and chitosan; it is distributed in almost all human tissues, highly concentrated in the connective tissues of the human body, and found at its highest concentrations in the cartilage [1]. GAH can synthesize mucopolysaccharides, glycoproteins, and proteoglycans, especially the intermediates for the synthesis of articular cartilage and synovial fluid molecules. It is endogenous biosynthesis in animals and humans by glucosamination. GAH has been shown to have a variety of biological functions, including anti-inflammatory, antioxidant, and anti-tumor activities [2,3,4]. Our previous work has demonstrated that *Agaricus bisporus*-derived GAH regulates the bone morphogenetic protein (BMP) signaling pathway to repair skeletal damage in zebrafish [5]. Due to its excellent anti-inflammatory and bone loss-promoting effects, GAH and its derivates have been utilized in dietary supplements and for therapeutic development [6,7]. However, there are few studies on the application of GAH in vascular disease, as its role in angiogenesis is not yet clear. Recent research reported that BMPs promote angiogenesis by stimulating the production of osteoblast-derived vascular endothelial growth factor A (VEGF-A) [8,9]. In our previous research, we found some clues that GAH has an impact on angiogenesis. The purpose of this study is to investigate the vascular repair mechanism of GAH to test the hypothesis that GAH promotes angiogenesis by regulating BMP signaling.

Zebrafish are an excellent vertebrate model in which to study vascular development as they are genetically similar to mammalian systems, demonstrate comparable molecular regulation of angiogenesis [10], and their embryos are small with high single spawning numbers, short developmental cycles, and transparency, which allows for easy observation [11,12]. Further, these characteristics make zebrafish an attractive system for high-throughput screening as they allow for straightforward observation of complex cellular behavior following administration of bioactive compounds in an intact vertebrate organism [13,14]. Several transgenic zebrafish strains expressing fluorescent proteins specifically in blood vessels have been previously developed and applied, including *Tg(fli1: GFP)*, *Tg(kdrl: mCherry)*, and *Tg(fli1: nGFP). Kdrl*, also named *Vegfr2*, is mainly expressed in the brain, cardiovascular, and hematopoietic systems of zebrafish and is involved in angiogenesis by binding VEGFs such as VEGFA to transduce downstream signaling pathways. *Fli1* is expressed in several structures, including the cardiovascular system, hematopoietic system, mesoderm, and pharyngeal arch, and is predicted to enable DNA-binding transcription factor activity and act upstream of or within angiogenesis [15,16,17]. These transgenic zebrafish with specific fluorescent markers can be traced using confocal microscopy, which allows for the precise observation of development and changes in blood vessels across time and space. These techniques can then be applied to study the effect of drugs on vascular defects [18,19,20].

Vascular endothelial growth factor (VEGF) is primarily appreciated as a potent angiogenic and vascular permeability-enhancing factor [21]. In mammals, the VEGF family consists of five secreted proteins: VEGF-A, B, C, and D, and placental growth factor (PLGF), which have different binding affinities for the three tyrosine kinase receptors VEGFR1, 2, and 3 [22]. VEGF signaling is recognized as one of the most important signaling pathways during angiogenesis and plays an important role in vascular development and disease [23,24]. Vascular endothelial growth factor receptor inhibitor (VRI) specifically inhibits VEGFR2, which in turn blocks VEGF signaling and angiogenesis, resulting in defective vascular development, and can be used to construct a vascular impairment model [25]. BMPs constitute the largest subdivision of the transforming growth factor-β family of ligands [26]. Defects in the BMP pathway or its regulation underlie multiple human diseases of different organ systems, including vascular developmental defects [27,28]. DMH1 and Dorsomorphin (DM) are inhibitors that selectively inhibit the BMP type I receptors; DMH1 targets ALK2, and DM binds to ALK2, ALK3, and ALK6, thereby inhibiting BMP signaling cascades as well as their downstream targets [29,30]. In this study, we used these inhibitors to construct zebrafish vascular-impaired models in a vascular-specific labeled fluorescent background to investigate the mechanism of blood vessel repair by GAH. We measured vessel fluorescence, mRNA expression, and protein levels to provide an experimental basis for screening safer and more effective drugs to repair vascular lesions.

## 2. Materials and Methods

### 2.1. GAH and Inhibitors

GAH was extracted from *Agaricus bisporus* in our lab according to previously published methods [5]. VRI, DMH1, and Dorsomorphin (DM) [29] were purchased from Merck and Sigma, respectively.

### 2.2. Transgenic Lines

The transgenic lines *Tg(kdrl: mCherry)* [15], *Tg(fli1: nGFP)* [17], and *Tg(BRE: GFP)* [31] were used in this study. They were gifted by Tsinghua University or purchased from the China Zebrafish Resource Center. All fish were raised and kept at 28.5 °C in the standard circulating water system with a 10/14 h dark/light cycle. Zebrafish embryos were obtained by natural spawning. All embryos and larvae were cultured in Holfreter H_2_O containing 3.5 g/L NaCl, 0.05 g/L KCl, 0.1 g/L CaCl_2_, and 0.025 g/L NaHCO_3_.

### 2.3. Drug Treatment

To observe the promoting effect of GAH on the vascular development of zebrafish, as mentioned in Section 2.2, embryos before 0.75 hpf (one-cell stage) were divided into four groups, namely, the control group, 0.1% (0.1 g/100 mL) GAH, 0.2% GAH, and 0.3% GAH treatment groups. After incubation for 24 or 48 h, embryos were collected for *in vivo* fluorescence observation (48 hpf), BrdU staining (48 hpf), or *in situ* hybridization (24 and 48 hpf).

VRI was used to block VEGF signaling in this study. In this experiment, embryos of *Tg(kdrl: mCherry)* at 24 hpf were divided into a control group, always cultured with Holfreter water; a modeling group treated with 0.15 mM VRI from 24 to 72 hpf; and recovery groups, which were treated with 0.15 mM VRI at 24 hpf and then replaced with Holfreter water, 0.1% GAH or 0.3% GAH at 48 hpf, subsequently cultured to 72 hpf for observation of fluorescent protein expression.

BMP signaling was suppressed by the specific inhibitors DMH1 and DM. Double transgenic embryos of *Tg(BRE: GFP; kdrl: mCherry)* at 24 hpf were treated with 10 μM DMH1 or 10 μM DM for continuous disrupting BMP transduction. In addition, in the presence of DMH1 and DM, 0.1% and 0.3% GAH were added for recovery, namely, 24 h + DMH1 and 0.1% GAH, 24 h + DMH1 and 0.3% GAH, 24 h + DM and 0.1% GAH, and 24 h + DM and 0.3% GAH. Moreover, a separate 0.3% GAH treatment group was set at 24 hpf, that is, 24 h + 0.3% GAH. All these groups were collected at 48 hpf for subsequent experiments.

### 2.4. BrdU Staining and WISH

Embryos of *Tg(fli1: nGFP)* were exposed to different concentrations of GAH solutions, as mentioned in Section 2.3, when cultured to 47.5 hpf, incubated with BrdU (10 mM) on ice for half an hour after protease membrane removal, and fixed with 4% paraformaldehyde (PFA) at 48 hpf. Then the BrdU incorporation assay was performed as previously described [32] to investigate the proliferation activity of vascular endothelial cells. BrdU and its antibody (source: mouse) were purchased from Sigma and Santa Cruz, respectively. GFP antibody was purchased from Abways (source: rabbit). BrdU antibody and GFP antibody were mixed, and the dilution ratio of each antibody was 1/300.

Whole-mount *in situ* hybridization (WISH) of zebrafish embryos was performed as described previously [33] using probes for the target *kdrl*. Embryos were observed using fluorescence stereomicroscope (Nikon, SMZ18).

### 2.5. Quantitative PCR and ELISA

Total RNA and cDNA were prepared according to our previous work [34]. Quantitative PCR was then performed using the SYBR green mix (powerup) to examine the relative expression levels of *bmp2b*, *bmp4*, *vegfaa, kdrl,* and *fli1*. The amplification program used a two-step method, and the fold change of these genes was calculated by QuantStudio™ Real-Time PCR Software v1.3. Three technical replicates were set for each gene in each group; the results were obtained based on three biological replicates, and *gapdh* was used as a reference gene.

Embryos under different treatments mentioned in Section 2.3 were collected at the required stage for the ELISA experiment, with 20 embryos in each group. Protein homogenate samples were obtained through lysing embryos, following an ELISA assay according to the standard experimental procedures provided by the kit. ELISA kits for Bmp2b, Bmp4, Vegfa, and Vegfr2 were purchased from Zgenebo (Taiwan, China) and eβios, which can specifically recognize zebrafish, and procedures were adopted from the respective kit. After the sample was reacted according to the kit procedure, the Optical Density (O.D.) was read at 450 nm, and then the corresponding protein amount of the sample was calculated from the standard curve. Primers for qPCR and protocols for ELISA used in this study can be provided upon request.

### 2.6. Statistical Analyses

**Figure 2:**
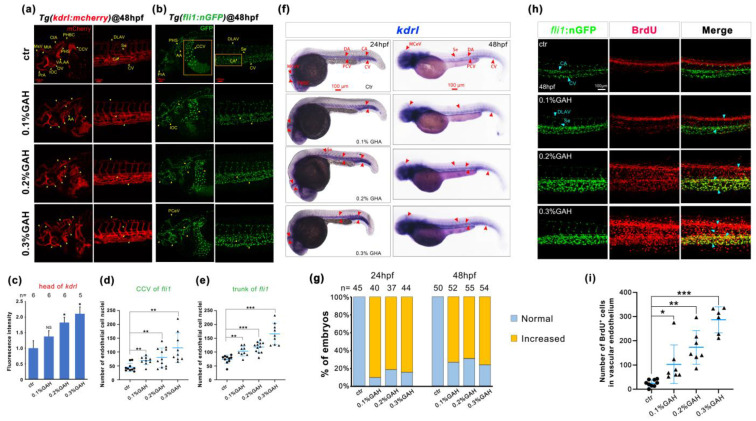
GAH promotes zebrafish vascular development. (**a**,**b**) Vascular development status of transgenic fish with labeled vascular endothelial cells (red) and their nuclei (green) after treatment with different concentrations of GAH as indicated at 48 hpf using a laser scanning confocal microscope. Yellow arrows indicated: CtA, central arteries; PHBC, primordial hindbrain channel; MsV, mesencephalic vein; MtA, metencephalic artery; PHS, primary head sinus; VA, ventral aorta; AA, branchial arch; IOC, inner optic circle; OV, optic vein; PrA, prosencephalic artery; Se, intersegmental vessel; DLAV, dorsal longitudinal anastomotic vessel; CCV, common cardinal vein; CA, caudal artery; CV, caudal vein; PCeV, posterior (caudal) cerebral vein. Column 1 (vertical row) was a diagram of the blood vessels in the head region; Column 2 was a diagram of intersegmental vessels. Column 3 was a diagram of the nuclei of the vascular endothelial cells in the head; Column 4 was a diagram of the intersegmental vessels’ vascular endothelial cell nuclei. The experiment was repeated two times. (**c**–**e**) Statistical data of (**a**,**b**). (**f**) Expression of *kdrl* at 24 hpf and 48 hpf by ISH with increasing concentrations of GAH as indicated. Red arrows indicated MCeV, middle cerebral vein; PMBC, primordial midbrain channel; DA, dorsal aorta; CA, caudal artery; PCV, posterior (caudal) cardinal vein; CV, caudal vein; Se, intersegmental vessel. (**g**) Statistical data of (**f**) based on three (24 hpf) and four (48 hpf) repetitions. (**h**) BrdU incorporation assay to investigate cell proliferation activity. Green fluorescence indicated a vascular endothelial nucleus; red fluorescence indicated BrdU-positive cells. The experiment was repeated twice. Scale bars: 100 μm. (**i**) Statistical data of the number of BrdU-positive cells co-located with GFP in (**h**). *p* < 0.05 (*), *p* < 0.01 (**), and *p* < 0.001 (***); NS, nonsignificant.

**Figure 3:**
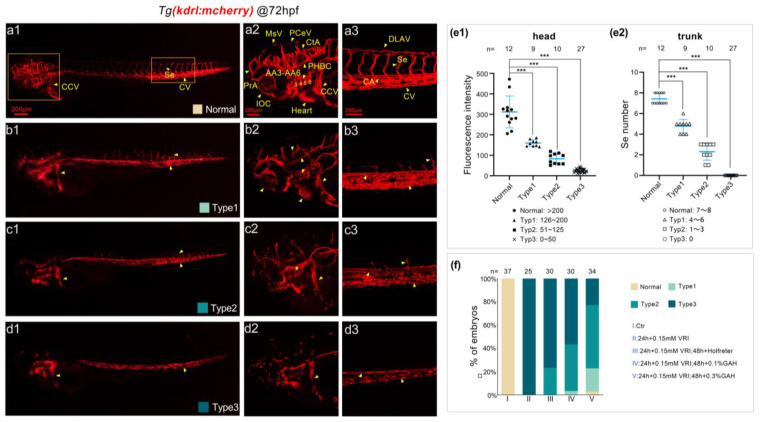
GAH repairs vascular impairment by regulating the VEGF signal. (**a1**–**d3**) The vascular development of *Tg(kdrl: mCherry)* zebrafish at 72 hpf under a laser scanning confocal microscope. Zebrafish embryos were exposed to 0.15 mmol/L VRI to induce vascular impairment and then treated with GAH for recovery. Type 1~Type 3 indicated different vascular impairment phenotypes, from slight to severe. Arrows indicated: Se, intersegmental vessel; CCV, common cardinal vein; CV, caudal vein; MsV, mesencephalic vein; PCeV, posterior cerebral vein; CtA, central artery; PrA, prosencephalic artery; IOC, inner optic circle; AA3–AA6, first to fourth branchial arch; PHBC, primordial hindbrain channel; DLAV, dorsal longitudinal anastomotic vessel; CA, caudal artery. (**a1**–**d1**): diagram of whole-body blood vessels. (**a2**–**d2**): enlarged diagram of the blood vessels in the head. (**a3**–**d3**): enlarged diagram of intersegmental vessels. (**e1**,**e2**): classification criteria and statistics of different types for head and trunk. *p* < 0.001 (***). (**f**) Statistical data of different types of vascular phenotypes across different treatment groups. The experiment was repeated three times.

**Figure 4:**
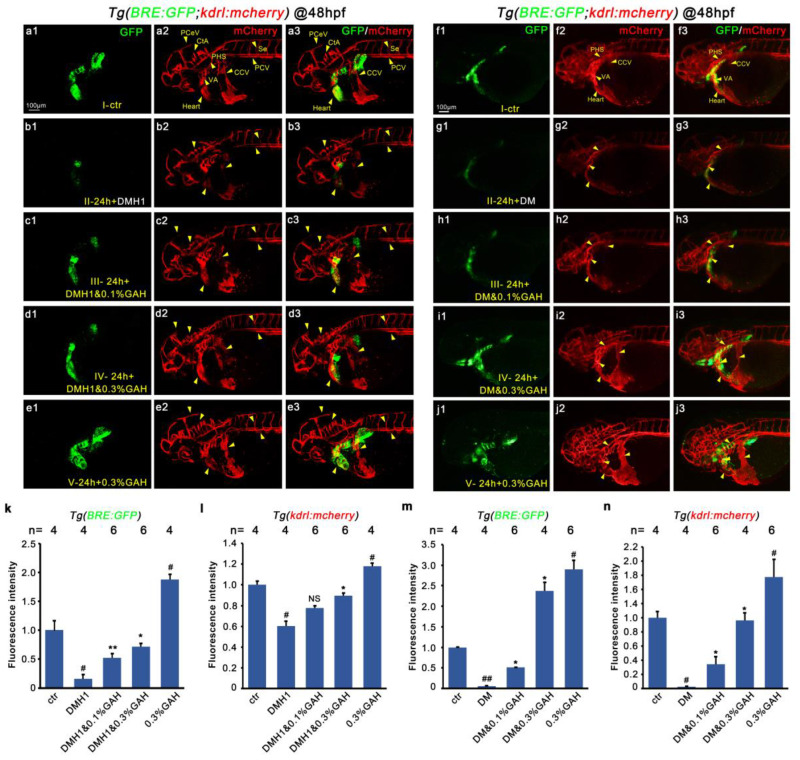
GAH restores BMP signaling and facilitates the repair of vascular impairment induced by DMH1 and DM. *Tg (BRE: GFP; kdrl: mCherry)* double transgenic embryos were was treated with 10 μM DMH1 (**a1**–**e3**) or 10 μM DM (**f1**–**j3**) and then treated with GAH as indicated. BMP (green) activity and vascular development (red) and their colocalization were observed by confocal at 48 hpf. Arrows indicated: Se, intersegmental vessel; MsV, mesencephalic vein; PCeV, posterior cerebral vein; CtA, central artery; PCV, posterior cardinal vein; PHS, primary head sinus; VA, ventral aorta; CCV, common cardinal vein. The experiment was repeated twice for each inhibitor. (**k**,**l**) Statistical data of BRE and mCherry fluorescent intensity after DMH1 inhibition (**a1**–**e1**) and (**a2**–**e2**). (**m**,**n**) Statistical data of (**f1**–**j1**,**f2**–**j2**). Compared with the ctr group, *p* < 0.05 (#), *p* < 0.01 (##); compared with the 24 h + 10 μM DMH1/DM group, *p* < 0.05 (*), *p* < 0.01 (**), and nonsignificant (NS).

The fluorescence intensity in Figure 2, Figure 3 and Figure 4 was calculated using Image J, taking a certain area of the zebrafish (the selected area of each fish was the same), and the fluorescence intensity of mCherry or GFP multiplied by the expression area was selected to represent the total intensity of each embryo. The number of embryos counted in each group was marked in the figure, and we used the average value as the fluorescence intensity of this group. The number of samples calculated for each group was based on the number of points in scatter diagrams (Figure 2d,e,i, and Figure 3e). Specifically, for calculating the number of endothelial cells in *Tg(fli: nGFP)* in Figure 2b, the regions selected for calculation were CCV distributed in the yolk and CA, CV regions located directly below the 5 somites after yolk extension (seen in the yellow box). The counting regions of BrdU+ cells co-located with GFP were all blood vessels seen in the images in Figure 2h, spanning the width of approximately 9 somites from the yolk extension, not only including all CAs and CVs within this range but also the DLAV and Se regions.

All data were presented as mean ± SD. In quantitative PCR and ELISA assays, the SD value was based on the results of biological replication experiments. All statistical analyses were performed using the GraphPad Prism 9 software. Student’s *t*-tests (two-tailed, unequal variance) were used to determine *p*-values for all measurements in this study. Comparisons with a *p*-value of *p* < 0.05 (*), *p* < 0.01 (**), and *p* < 0.001 (***) were considered statistically significant.

## 3. Results

### 3.1. HPLC and ^1^HNMR Analysis of Agaricus bisporus GAH

**Figure 1:**
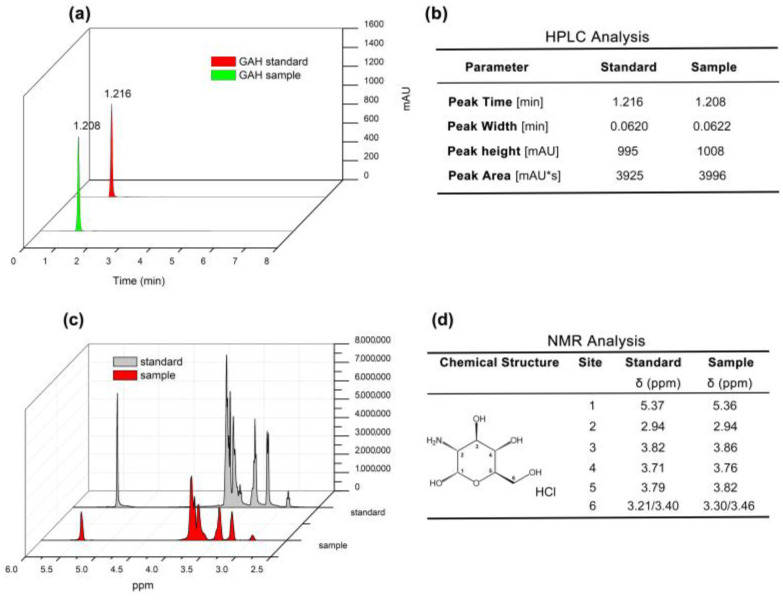
HPLC and ^1^HNMR data of GAH. (**a**) Peak graph of standard and *Agaricus bisporus*-derived (sample) GAH. (**b**) Related parameter of (**a**). (**c**) Proton NMR spectra. (**d**) Chemical shifts of the ^1^H of standard and sample GAH.

The glucosamine hydrochloride (GAH) component was successfully extracted and purified from the cell wall of *Agaricus bisporus* via a known process [5]. High-performance liquid chromatography (HPLC) was performed, and the results showed that the peak time of *Agaricus bisporus*-derived GAH was the same as that of the standard sample, which appeared in 1.208 min and 1.216 min, respectively, and both were single peaks with very close peak heights and peak widths. Interestingly, the peak area of 2 mg/mL *Agaricus bisporus* GAH was slightly higher than that of the standard GAH, with a peak area ratio of 1.018 (3996/3925), suggesting that the purity of *Agaricus bisporus* GAH was higher (Figure 1a,b). Furthermore, the chemical shifts were detected by ^1^H spectrum nuclear magnetic resonance (^1^HNMR), and the data showed that the chemical shifts of the H protons on the six carbon atoms in the sample GAH were consistent with those in the standard GAH (Figure 1c,d). Based on the above experimental results, the substance isolated from *Agaricus bisporus* in this study is glucosamine hydrochloride (GAH).

### 3.2. GAH Promotes Vascular Development in Zebrafish

One-cell stage of *Tg(kdrl: mCherry)*, *Tg(fli1: nGFP)* embryos were cultured in 0.1%, 0.2%, and 0.3% GAH solutions according to a semi-lethal assay [5], and vascular development was observed using a laser scanning confocal microscope at 48 h post-fertilization (hpf). Expression of mCherry was gradually increased with increasing GAH concentrations, including enhancement in fluorescence intensity and expression area, such as in the common cardinal vein (CCV), central arteries (CtA), primordial hindbrain channel (PHBC), mesencephalic vein (MsV), metencephalic artery (MtA), primary head sinus (PHS), ventral aorta (VA), branchial arch (AA), inner optic circle (IOC), optic vein (OV), intersegmental vessel (Se), dorsal longitudinal anastomotic vessel (DLAV), and caudal artery (CA). In the 0.2% and 0.3% GAH groups, there was significant hyperplasia in the VA, AA, CCV, CA, and Se regions. Vascular development was the most abundant, and fluorescence was the strongest when the embryos were treated with 0.3% GAH. Statistical analysis of fluorescence intensity showed that the GAH-induced fluorescence was higher than the control group across all concentrations, with significant differences at concentrations of 0.2% and 0.3% GAH groups (Figure 2a,c). Moreover, the number of nuclei in vascular endothelial cells of *Tg(fli1:nGFP)* was also gradually increased after GAH treatment in different concentrations, specifically in the CCV, CA, and CV (marked with a yellow box), indicating vascular endothelial cell proliferation (Figure 2b,d,e).

Consistent with the vascular development phenotype, the results of *in situ* hybridization (ISH) revealed that GAH treatment upregulated the expression of the vascular marker gene *kdrl*. The expression of *kdrl* in MCeV, PCV, dorsal aorta (DA), caudal vein (CV), and Se was gradually enhanced with the increase of GAH concentration, and this enhanced expression can persist from 24 hpf to 48 hpf (Figure 2f). Statistical analysis showed that at these two developmental stages, the proportion of embryos with increased expression of *kdrl* exceeded 65% (Figure 2g). In addition, BrdU incorporation was furtherly performed to detect cell proliferation, BrdU-positive cells had a pronounced increase at DLAV, CA, and CV, and colocalized signals of red and green fluorescence at the above sites were significantly increased in the 0.3% GAH group (Figure 2h). Statistical analysis showed that BrdU-positive cells in the vascular endothelium gradually increased in a GAH dose-dependent manner, implying the promoting effect of GAH on vascular endothelial cell proliferation (Figure 2h,i). Taken together, these results suggest that GAH promotes the proliferation of vascular endothelial cells by upregulating vascular development regulatory genes, thereby promoting vascular development.

### 3.3. GAH Promotes Vascular Development through VEGF Signaling

Previous work has suggested that GAH promotes vascular development by upregulating the expression of vascular-specific markers and promoting the proliferation of vascular endothelial cells. We speculate that GAH may be involved in VEGF signaling regulation. To investigate this, *Tg(kdrl: mCherry)* embryos were cultured to 24 hpf and divided into control (group I) and modeling groups (group II); the control group was incubated with Holfreter water, and the modeling group was incubated with 0.15 mmol/L VRI, which specifically inhibits the tyrosinase type II receptor of the VEGF signaling pathway and induces vascular impairment. At 48 hpf, the VRI-treated embryos were divided into the VRI continuous modeling group (group II), the negative control group (group III), replacing VRI with Holfreter water, and the 0.1% and 0.3% GAH recovery groups (group IV and group V, respectively), and cultured to 72 hpf. Treatment with VRI caused serious vascular developmental impairment, including reduction or loss of Se, head vessels, and eye vessels, as well as thinning of the CV. We classified the vascular developmental phenotypes of five different groups and statistically analyzed the proportion of different phenotypes in different groups. As shown in Figure 3, according to the fluorescence intensity of the head and the number of intersegmental vessels (approximately seven segments after the yolk extension), the vascular developmental phenotypes were divided into four categories (Normal, Type 1~Type 3). Normal meant that the vascular development was consistent with the wild type, the fluorescence intensity of the head was more than 200, and the number of Se in the framed area (yellow box) was 7–8 (Figure 3(a1–a3)). Type 1 was classified as having a slight impairment, with thinning or reducing of blood vessels in the head, such as in the PrA, CtA, AA, PHBC, and CCV regions, and incomplete development of Se. The fluorescence intensity of this type in head blood vessels ranged from 126 to 200, and the number of Se was 4–6 (Figure 3(b1–b3)); Type 2 was a moderately impaired type, with further weakening or loss of head vascular development in the aforementioned areas, especially periocular vessels. There was a severe loss of intersegmental vessels, with a sharp decrease in number in Type 2. The fluorescence intensity range of the head was 51 to 125, and the number of Se in specific areas was 1–3 (Figure 3(c1–c3)); Type 3 represented the most serious impairment, with almost invisible or complete loss of blood vessels between the head and body segments, with a fluorescence intensity range of 0–50 in the head and a Se number of 0 in specific regions (Figure 3(d1–d3)). Compared with normal embryos, there were significant differences in different types of vascular impaired phenotypes (Figure 3(e1,e2)).

Furtherly, the repaired effects of different drug recovery groups on vascular development were analyzed through a percentage stacking diagram. We found that VRI treatment (group II) led to the most severe vascular development phenotype (Type3), with a proportion of 100%. While the recovery groups treated with GAH or Holfreter water exhibit different degrees of vascular developmental impaired phenotypes and varying proportions. There were approximately 77% of Type 3 and 23% of Type 2 phenotypes in group III, indicating a natural, modest repair of vascular impairment when recovered by Holfreter water. In the 0.1% GAH group (group IV), the proportion of Type 3 was reduced to 57%, and phenotypes of Type 1 and Type 2 appeared, with a proportion of 3% and 40%, respectively, indicating that GAH can significantly alleviate vascular development defects, but Type 3 was still the dominant phenotype. While in the 0.3% GAH group (group V), the proportion of Type 3 dropped sharply from 100% to only 23%, both Type 1 and Type 2 phenotypes were raised to 20% and 54%, respectively. In this group, even 3% of the larvae returned to the normal phenotype in vascular development (Figure 3f). These results suggest that GAH may repair vascular developmental impairment and promote vascular development through the VEGF signaling pathway.

### 3.4. GAH Repairs Vascular Impairment Caused by DMH1 and DM

The data above have demonstrated that GAH can regulate the VEGF signaling pathway to promote vascular impairment repair. A recent study reported that VEGF is regulated by BMP signaling [35]. Because we have previously found that GAH can regulate the BMP signaling pathway to repair skeletal injury in zebrafish [5], we wanted to investigate whether GAH regulates VEGF through BMP signaling to repair vascular impairment. To this end, two kinds of BMP-specific inhibitors were used in this study. DMHl can specifically bind to Alk2, and dorsomorphin (DM) can specifically bind to Alk2/3/6 and AMPK [30].

Double transgenic embryos of *Tg(BRE: GFP; kdrl: mCherry)*, with GFP characterizing BMP signal activity and mCherry representing VEGF, were collected and divided into five groups: Group I, the control group, incubated with Holfreter water; Group II, the modeling group, was incubated with Holfreter water until 24 hpf and then replaced with 10 μM DMH1; Group III was incubated with Holfreter water until 24 hpf and replaced with 10 μM DMH1 + 0.1% GAH; Group IV was incubated with Holfreter water until 24 hpf and replaced with 10 μM DMH1 + 0.3% GAH; and Group V was incubated with Holfreter water until 24 hpf and replaced with 0.3% GAH. Embryos in different groups were cultured to 48 hpf to observe the expression of two fluorescent proteins under a laser confocal microscope.

As shown in Figure 4, DMH1 treatment led to significantly reduced GFP expression compared to the control group, suggesting that the BMP signal was suppressed (Figure 4(a1,b1)). Meanwhile, the mCherry vascular fluorescence was also significantly weakened, particularly in the PCV, Heart, CtA, and Se regions; even the expression in PCeV and CCV almost disappeared, implying that disruption of BMP signaling may cause vascular developmental impairment (Figure 4(a2,b2)). The decreased GFP fluorescence was recovered by GAH treatment in a dose-dependent manner (Figure 4(c1,d1)). This GAH-dependent recovery was also observed for mCherry, such as the restored expression in CCV, CtA, Heart, Se, and PCV when recovered by 0.3% GAH (Figure 4(c2,d2)), suggesting that GAH can specifically repair the weakened BMP and VEGF signals caused by DMH1. In addition, the colocalization of BMP and VEGF signals showed that mCherry fluorescence was significantly restored in the same regions where the GFP fluorescence was recovered, such as the VA, CCV, and Heart (Figure 4(c3,d3)). Moreover, colocalization of mCherry and GFP was greatly enhanced when treated with 0.3% GAH alone compared to the control group (Figure 4(e1–e3)). The fluorescence intensity of GFP and mCherry were statistically analyzed, consistent with the observed changes in fluorescence expression. The fluorescence intensity of GFP was significantly reduced after being treated by DMH1, as was that of mCherry, among which GFP decreased to about 15% of the control group and mCherry intensity decreased to about 60% of the control group. GAH showed a dose-dependent effect on the recovery of fluorescence intensity of GFP and mCherry, in which the GFP in the GAH-recovered group increased 3 to 4-fold compared with the model group (Group II). In addition, the mCherry intensity of embryos in the GAH-only treated group (Group V) was significantly higher than that of the control group (Figure 4k,l). The data above furtherly support the possible regulatory role of BMP on VEGF during vascular development.

Using the same grouping method as DMH1, five groups of embryos were collected at 48 hpf to observe the GFP and mCherry expression after treatment with another inhibitor, DM, and found a similar trend as DMH1. In the DM group, all of the embryos showed decreased expression of GFP and mCherry, specifically, the cerebral and ocular blood vessel signals were sharply reduced and the intersegmental vessels were nearly invisible (Figure 4(f1,f2,g1,g2)). GAH had a significant repaired effect on both BMP and vascular defects; specifically, in the DM + 0.3% GAH group, the green fluorescence but not mCherry (VEGF) was even stronger than the control group, along with the restoration of red fluorescent protein expression at the CCV, VA, PHS, and Heart. In addition, GFP and mCherry have a significant colocalization expression in these restored expression regions (Figure 4(h1–h3,i1–i3)). The statistical analysis results showed that the decrease in GFP and mCherry intensity caused by DM was even more significant than that caused by DMH1. Both GFP and mCherry decreased to around 5% of the control, and 0.1% GAH can greatly restore GFP and mCherry fluorescence intensities, both exceeding 10 folds. When the GAH concentration increased to 0.3%, the intensity of mCherry almost returned to the level of the control group; surprisingly, the fluorescence intensity of GFP in this group even reached about 2.3 folds that of the control group (Figure 4m,n), indicating that in the model of GAH repairing vascular impairment induced by DM, the effect of DM on AMPK may not be significant, and GAH mainly plays a role through the recovery of BMP. The above results indicate that inhibiting BMP signaling can cause VEGF dysfunction, leading to vascular development impairment, and GAH can promote vascular impairment repair through BMP signaling.

### 3.5. GAH Regulates VEGF to Repair Vascular Impairment through BMP Signaling

**Figure 5:**
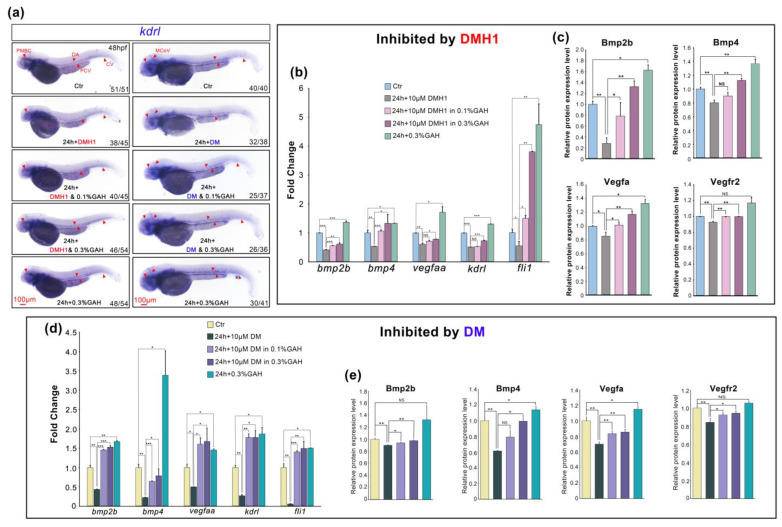
GAH repairs vascular impairment by upregulating the mRNA and protein levels of BMP and VEGF signaling pathway members. (**a**) Spatiotemporal expression of *kdrl* by ISH in different groups. Red arrows indicated the specific expression of *kdrl* in PMBC, PCV, CV, DA, and MCeV. Scale bars: 100 μm. The proportion of embryonic expression patterns in each group was shown as indicated and based on four (for DMH1) and three (for DM) repetitions. (**b**,**d**) Relative mRNA expression levels of *bmp2b*, *bmp4*, *vegfaa*, *kdrl,* and *fli1* by quantitative PCR in different treatment groups as indicated. *gapdh*: reference gene. Each gene was tested at least five times. (**c**,**e**) Protein levels of Bmp2b, Bmp4, Vegfa, and Vegfr2 in different groups by ELISA assay. Each protein was repeated 2–3 times. *p* < 0.05 (*), *p* < 0.01 (**), *p* < 0.001 (***), and NS, nonsignificant.

The above results demonstrated that GAH is involved in VEGF signal regulation and that the colocalization of BMP and VEGF is enhanced by GAH after disruption by DMH1 or DM, indicating that GAH may regulate VEGF through BMP signaling. To verify this hypothesis, we measured the transcriptional and protein levels of members of these two signaling pathways after blocking BMP by DMH1 and DM with or without GAH recovery, which was the same as the previous treatment. We firstly examined the spatiotemporal mRNA distribution of *kdrl* at 48 hpf by ISH. DMH1 led to a remarkably reduced expression of *kdrl* in both intensity and range. Especially in PMBC, PCV, CV, DA, and cerebral blood vessels, the staining signals in these regions became barely visible. While GAH can significantly restore the weakened expression of *kdrl* in the above areas caused by DMH1, the restoration even returned to the control level in the 0.3% GAH group. The staining intensity of *kdrl* in both the head and trunk blood vessels was significantly enhanced compared to the control group following treatment with 0.3% GAH alone. Expression of *kdrl* showed a similar trend of change after DM inhibition (Figure 5a). In addition, quantitative PCR was also performed to detect the fold changes in the mRNA expression levels of BMP and VEGF members. Consistent with *in situ* hybridization results, both DMH1 and DM inhibition significantly downregulated the expression levels of *bmp2b*, *bmp4*, *vegfaa*, *kdrl*, and *fli1*, and these decreased expressions can be upregulated by different concentrations of GAH. The recovery in the 0.3% GAH group was better and even exceed that of the control group. Meanwhile, the results of both BMP disruption experiments using DMH1 and DM showed that 0.3% GAH treatment alone could promote mRNA expression levels of BMP and VEGF members (Figure 5b,d). Finally, the protein levels of members of the BMP and VEGF signaling pathways were detected by an ELISA assay. After disrupting BMP signaling with DMH1 or DM, Bmp2b, Bmp4, Vegfa, and Vegfr2 protein levels were distinctly decreased. 0.3% GAH can significantly restored all downregulation caused by DMH1 or DM. Moreover, the protein expression levels of BMP and VEGF members treated with GAH alone were all higher than those of the control group, approximately upregulated to1.1-1.6 times that of the control group. (Figure 5c,e). These results suggest that GAH can repair vascular impairment and promote vascular development by upregulating the expression levels of genes related to the BMP and VEGF signaling pathways.

## 4. Discussion

Our other work has proved that GAH reduces blood sugar and repairs vascular lesions induced by diabetes. However, there are currently no reports on the roles of GAH in vascular development. In this study, transgenic zebrafish and specific inhibitors of VEGF and BMP signaling were used to explore the mechanism of GAH in vascular impairment repair. We firstly found that GAH from *Agaricus bisporus* can promote vascular development (Figure 2). In addition, we demonstrated that GAH can repair vascular developmental impairment induced by the VEGF-specific inhibitor VRI, indicating GAH may regulate vascular development through VEGF signaling and is not dependent on VEGFR (Figure 3). Furthermore, our previous work has well demonstrated that GAH activates BMP and acts downstream of the BMP receptor level [5]. In this study, we showed that GAH can rescue vascular impairment that occurs when the BMP signaling pathway is blocked using the inhibitors DMH1 and DM by observing the colocalization of BMP and VEGF signaling components and measuring their mRNA and protein levels (Figure 4 and Figure 5). Based on the above data, it is sufficient to suggest that GAH promotes angiogenesis by regulating VEGF signaling through BMP signaling.

**Figure 6:**
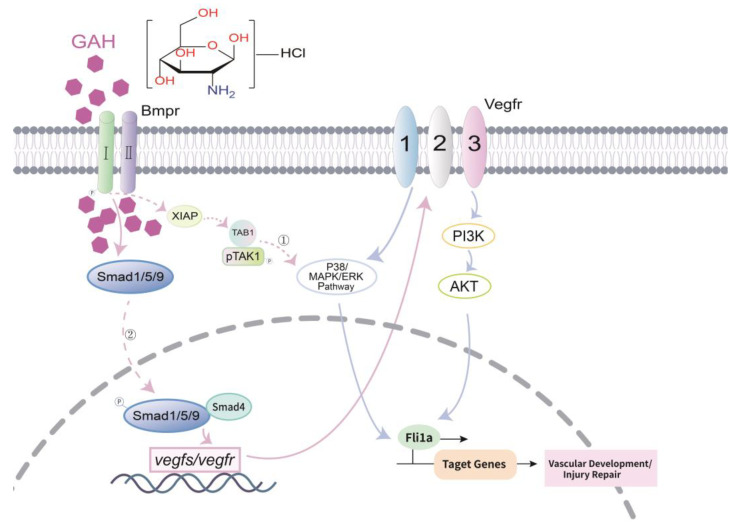
Working model of GAH-dependent vascular repair. In zebrafish, GAH activates BMP signaling transduction through non-Smad pathways to stimulate VEGF signaling or to activate the transcriptional levels of VEGF members via Smad1/5/9, which in turn promotes vascular development and impairment repair.

However, the intracellular mechanism of GAH is worth furtherly exploration. Previous studies have shown that noncanonical BMP signaling can crosstalk with VEGF to regulate angiogenesis through downstream signaling cascades such as the p38/MAPK/ERK pathways [36,37]. Recent studies have also reported that BMPs stimulate angiogenesis through the production of VEGF-A in the mouse preosteoblast-like cell line KS483. The angiogenic activity of VEGF can be amplified by combining with BMP2 [8,38,39]. In addition, BMP/Smad signaling may be involved in the transcriptional regulation of *VEGF*-related genes [35]. To this end, we analyzed the potential transcriptional regulation of BMP on the VEGF signaling pathway through the Promoter Prediction Analysis Website (http://jaspar.genereg.net/, URL (accessed on 17 November 2022)). We found that the promoter regions of zebrafish *vegfs and vegfrs* all contained converse binding sites of Smad4 and Smad5. Among them, *vegfaa*, *vegfab,* and *vegfr2* showed a relatively high binding possibility (Table S1), consistent with their expression changes in the process of vascular impairment and repair (Figure 4 and Figure 5), suggesting the BMP signal may directly target VEGF by activating their transcriptional levels via Smad4/5 binding. Taken all together, we propose two possible working hypotheses for GAH in cells, as shown in Figure 6. One is that GAH enters the cell to activate BMP type I receptors and the downstream non-Smad signaling pathway to activate VEGF; another way is to activate the transcription of downstream *VEGF* genes through BMP/Smad signaling, thereby promoting vascular development and repairing vascular impairment (Figure 6). The underlying regulatory mechanism between the GAH and BMP signal and its downstream target genes such as *vegfs/vegfr* remains unclear and should be addressed in subsequent experiments.

In summary, our experimental results provide a theoretical basis for the screening of effective drugs for the repair of vascular lesions and provide new research directions and support for GAH used as a drug in the treatment of vascular diseases and diabetic vascular complications.

## Data Availability

Data are available by contacting the corresponding author upon reasonable request.

## Supplementary Materials

The following supporting information can be downloaded at: https://www.mdpi.com/article/10.3390/life13122330/s1, Table S1: Analysis of Smads Binding to the Promoter Sequence of Zebrafish *Vegf* Genes.
