# Supplementary material for: Agaricus bisporus-Derived Glucosamine Hydrochloride Regulates VEGF through BMP Signaling to Promote Zebrafish Vascular Development and Impairment Repair
Source: Life (Basel). 2023 Dec 12;13(12):2330. doi: 10.3390/life13122330 (PMC10745105; doi:10.3390/life13122330)
Supplement: Supplementary file 1 [file life-13-02330-s001.zip › life-2586368-supplementary.pdf]

Table S1. Analysis of SMADs Binding to the Promoter Sequence of Zebrafish *Vegf* Genes.

Matrix ID/ Logo	Relative Score	Sequence ID	Target Region	Strand	Predicted Sequence
MA1557.1 SMAD 5 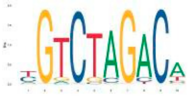	0.94891	vegfab	-4571～-4562	+	tgtctagcca
	0.90835	vegfr3	-521～-512	+	tgtctagccc
	0.87307	vegfaa	-3864～-3855	+	cgtcgagcct
	0.86974	vegfd	-1483～-1474	-	tgcctcgaca
	0.86920	vegfba	-5717～-5708	-	cgtccagtca
	0.84761	vegfr2	-3744～-3735	+	tatctagcca
	0.83856	vegfr1	-436～-427	+	taactagaca
	0.82700	vegfc	-358～-349	-	cggttagaca
MA1153.1 SMAD4 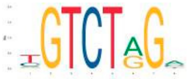	0.98806	vegfc	-4972～-4965	+	tgtctgga
	0.98179	vegfaa	-6523～-6516	-	tgtctagc
	0.98179	vegfab	-2904～-2897	+	tgtctagc
	0.98179	vegfr3	-521～-514	+	tgtctagc
	0.96219	vegfd	-416～-409	-	cgtctgga
	0.95476	vegfr1	-434～-427	-	tgtctagt
	0.94399	vegfba	-491～-484	-	cgtctggc
	0.91056	vegfr2	-4031～-4024	+	ggtctggc
	0.88722	vegfbb	-1411～-1404	-	ggtctggg
